# Mitochondrial uncoupling proteins and energy metabolism

**DOI:** 10.3389/fphys.2015.00036

**Published:** 2015-02-10

**Authors:** Rosa A. Busiello, Sabrina Savarese, Assunta Lombardi

**Affiliations:** ^1^Dipartimento di Scienze e Tecnologie, Università degli Studi del SannioBenevento, Italy; ^2^Dipartimento di Scienze e Tecnologie Ambientali, Biologiche e Farmaceutiche, Seconda Università degli Studi di NapoliCaserta, Italy; ^3^Dipartimento di Biologia, Università degli Studi di NapoliNapoli, Italy

**Keywords:** uncoupling protein, energy metabolism, mitochondria, proton-leak, obesity

## Abstract

Understanding the metabolic factors that contribute to energy metabolism (EM) is critical for the development of new treatments for obesity and related diseases. Mitochondrial oxidative phosphorylation is not perfectly coupled to ATP synthesis, and the process of proton-leak plays a crucial role. Proton-leak accounts for a significant part of the resting metabolic rate (RMR) and therefore enhancement of this process represents a potential target for obesity treatment. Since their discovery, uncoupling proteins have stimulated great interest due to their involvement in mitochondrial-inducible proton-leak. Despite the widely accepted uncoupling/thermogenic effect of uncoupling protein one (UCP_1_), which was the first in this family to be discovered, the reactions catalyzed by its homolog UCP_3_ and the physiological role remain under debate. This review provides an overview of the role played by UCP_1_ and UCP_3_ in mitochondrial uncoupling/functionality as well as EM and suggests that they are a potential therapeutic target for treating obesity and its related diseases such as type II diabetes mellitus.

## Introduction

Mitochondria are the major regulators of cellular energy metabolism (EM). Alterations of mitochondrial functionality have been linked to the pathogenesis of some metabolic disorders, including obesity and type II diabetes mellitus (T2DM).

The principal function of mitochondria is ATP production. Reduced cofactors, such as NADH and FADH_2_, obtained from oxidizable molecules (carbohydrates, lipids, and proteins) supply electrons to the electron transport chain (ETC) and result in a final reduction of molecular oxygen to water. Electron transfer through the ETC controls pumping of protons (H^+^) from the matrix to the intermembrane space, thus creating a proton-motive force (Δ*p*), whose energy is used by ATP synthase for the phosphorylation of ADP.

**Figure 1:**
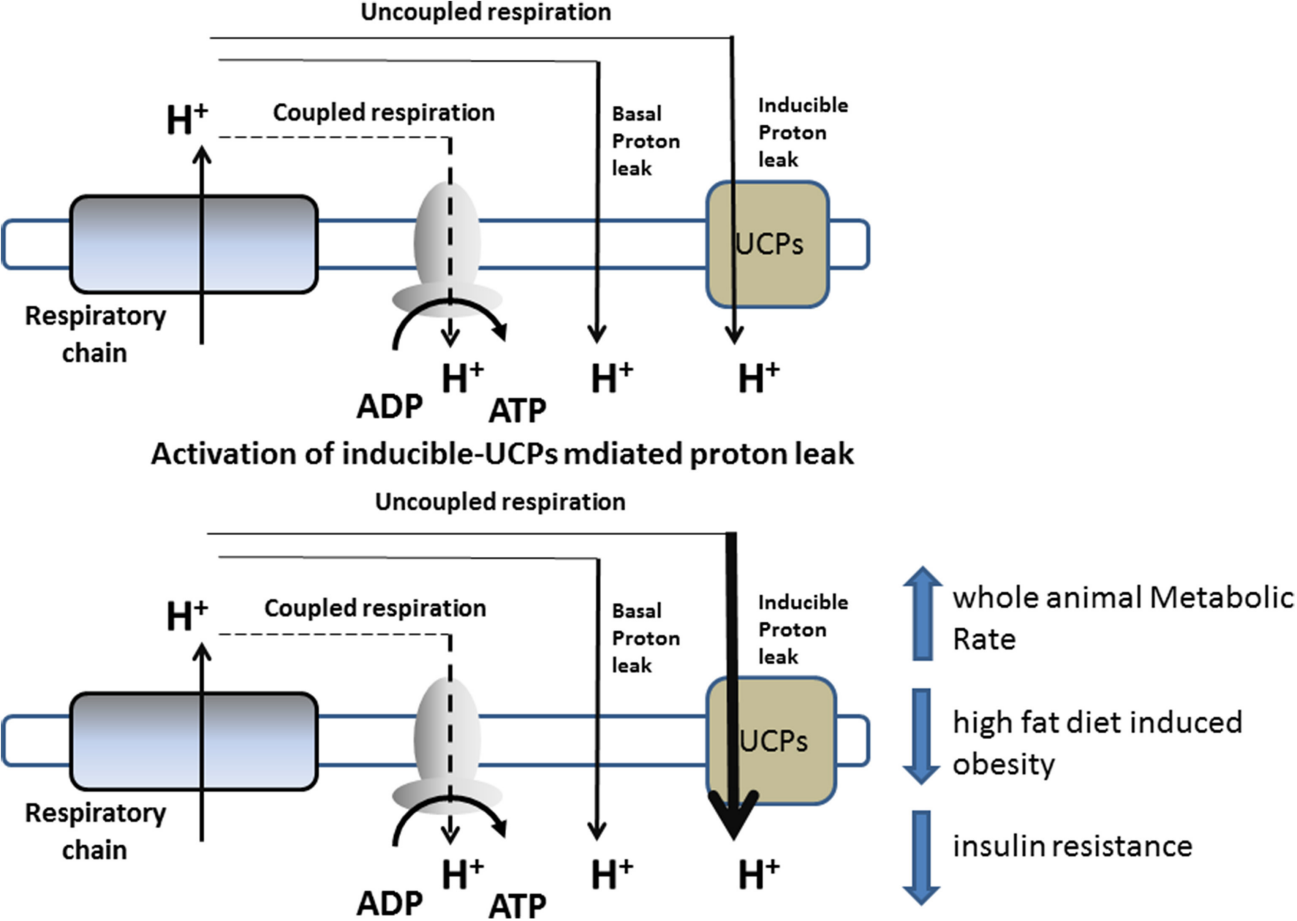
**Schematic representation of proton-leak and of the proposed role of UCPs in influencing energy metabolism**.

Mitochondrial oxidative phosphorylation is not perfectly coupled to ATP synthesis, since a portion of the energy liberated from the oxidation of dietary energy substrates is lost as heat instead of being converted into ATP. Indeed, some of the energy present in Δ*p* dissipates as heat by the re-entry of H^+^ into the matrix, through pathways independent of ATP synthase (proton-leak). Proton-leak is the sum of two processes: basal- and inducible proton-leak (Brand and Esteves, 2005). The first is not acutely regulated, but rather depends on the fatty-acyl composition of the mitochondrial inner membrane and the presence of adenine nucleotide translocase. On the other hand, inducible proton-leak is acutely controlled, with uncoupling proteins playing a crucial role (Divakaruni and Brand, 2011) (Figure 1).

Proton-leak has a marked influence on the entire EM of an organism, and, in rats, accounts for approximately 20–30% of the resting metabolic rate (RMR) (Rolfe and Brand, 1996). Variations in the proton-leak process contribute to the development of obesity or weight loss (Harper et al., 2008), and thus there has been increasing interest in targeting this process in order to increase RMR to treat obesity and other related diseases (Figure 1).

The present review discusses the relevance of UCPs on influencing EM. In particular, it focuses on uncoupling protein one (UCP_1_) and UCP_3_ due to their expression in brown adipose tissue (BAT) and skeletal muscle (SkM), respectively, which significantly contributes to EM.

## Uncoupling protein-1 (UCP_1_)

UCPs are members of the mitochondrial anion carrier family. The first uncoupling protein identified, UCP_1_, is predominantly expressed in BAT where it represents approximately 10% of the mitochondrial protein content and plays a thermogenic role through the catalysis of proton-leak. Recent evidence indicates the existence of two types of thermogenic adipocytes expressing UCP_1_: the classical brown adipocytes, which are found in the intercapsular brown adipose organ, and beige/bright adipocytes, which are found in subcutaneous WAT. Only recently it has unequivocally been proven that UCP_1_-positive cells found in WAT (i.e., beige adipocytes) are thermogenic-competent (Shabalina et al., 2013) and are actually a distinct subpopulation of white adipocytes that originate from a different lineage (reviewed in Harms and Seale, 2013).

One of the most prominent differences between brown and beige adipocytes is that brown cells express high levels of UCP_1_ and other thermogenic genes under basal (unstimulated) conditions, whereas beige adipocytes only express these genes in response to activators, such as β-adrenergic and peroxisome proliferator-activated receptor-γ agonists (Petrovic et al., 2010; Wu et al., 2013; Carrière et al., 2014). Nevertheless, both adipocyte types show comparable levels of UCP_1_ upon stimulation, indicating that under specific conditions they may have the same thermogenic capacity (Wu et al., 2012).

Cold and overfeeding are physiological stimuli that influence BAT thermogenesis through the enhancement of sympathetic overflow to BAT (Cannon and Nedergard, 2004). Acute sympathetic nerve activation stimulates heat production by activating UCP_1_ function. Indeed, noradrenergic stimulation of brown adipocytes activates cAMP-linked signaling pathways, which results in increased (i) mitochondria number and size, (ii) UCP_1_ gene transcription and translation, and (iii) UCP_1_ thermogenic activity through activation of lipolysis and release of acute regulators of UCP_1_, such as free fatty acids.

Prolonged cold exposure stimulates the proliferation and differentiation of brown precursor cells to expand BAT mass and increase thermogenic capacity (Bukowiecki et al., 1982). Moreover, it also induces beige adipocyte development and function (Vitali et al., 2012). Although sympathetic nerve activity was previously thought to be the major physiological signal that activates BAT thermogenesis and induces beige adipocyte development, numerous hormones and factors have now been shown to regulate such processes. Moreover, brown and beige adipocytes can be selectively recruited and activated (for review Cannon and Nedergard, 2004; Harms and Seale, 2013). The molecular mechanisms of UCP_1_ activation have been comprehensively discussed in other recent reviews and will not be elaborated upon here (Klingenberg and Echtay, 2001; Porter, 2008).

## Role of UCP_1_ in energy metabolism

The presence of UCP_1_ provides a marked capacity for BAT to dissipate energy by as much as 20%. In rodents, overeating activates BAT “diet-induced thermogenesis,” that preserve energy balance and contrasts obesity by reducing metabolic efficiency (Cannon and Nedergard, 2004). The overexpression of UCP_1_ or activation of BAT thermogenesis has been shown to prevent the development of obesity (Krauss et al., 2005). In contrast, the first experiments performed on UCP^−/−^_1_ mice housed at a standard temperature (20–22°C) failed to show an enhanced obesity predisposition compared to their wild-type counterparts (Enerback et al., 1997). More recently, Feldmann et al. (2009) reported that UCP_1_ ablation induced obesity in mice housed at 30°C, i.e., the thermoneutral temperature at which mice facultative thermogenesis is kept at a minimum. Indeed, when housed at 20–22°C, mice are under chronic thermal stress and inevitably increase their metabolic rate (~50%) to maintain body temperature (Golozoubova et al., 2006). Therefore, UCP^−/−^_1_ mice, that are unable to enhance BAT thermogenesis, have necessarily expend extra energy to defend their body temperature; the activation of alternative compensatory mechanisms, such as shivering thermogenesis, could have masked the role of UCP_1_ on EM. The study of Feldmann et al. (2009) also suggests that the impact of housing temperature on EM has been overlooked by most of the studies on animal models, and that cold stress could have confounded many studies. Humans tend to live at thermoneutrality, with the aid of clothing and heating. Thus, to translate the metabolic studies performed in mice to humans, experiments should be conducted under thermoneutral conditions.

Other evidence also support the role for UCP_1_ in EM. For example, transgenic mice expressing UCP_1_ in white fat depots display a lean phenotype (reviewed in Klaus et al., 2012). Moreover, muscle-specific ectopic expression of UCP_1_ leads to increased energy expenditure, delayed diet-induced obesity development, improved glucose homeostasis, increased insulin stimulated glucose uptake, and increased lipid metabolism (Keipert et al., 2013; Ost et al., 2014). This is in agreement with evidence showing that the selective induction of SkM UCP_1_-mediated proton-leak leads to an increased whole body energy expenditure as well as decreased adiposity (Adjeitey et al., 2013).

When chronically activated by cold exposure, UCP_1_ mediated-BAT thermogenesis enhances the oxidation of metabolic substrates necessary for sustaining enhanced thermogenesis. Under these conditions, BAT not only use stored lipids as substrates, but also large quantities of glucose and triglycerides (the last mainly in the form of chylomicrons) from the circulation (Bartelt et al., 2012; Peirce and Vidal-Puig, 2013). Therefore, by decreasing plasma lipids, lowering plasma glucose, and diminishing obesity, BAT has a potential role in protecting against obesity and T2DM.

## UCP_1_ expressing adipocytes are present in adult human

It was previously thought that in humans, BAT disappears rapidly after birth. However, more recently the use of radiodiagnostic techniques [positron emission tomography (PET)/computed tomography (CT)] together with histological methods has unequivocally identified the presence of UCP_1_-expressing adipocyte depots in adult humans. The tissue is not present in defined regions, but rather scattered within the WAT (Nedergaard et al., 2007). However, it is currently unclear whether the deposits of UCP_1_-expressing adipocytes in adult humans are analogous to beige or brown fat.

The enhancement of BAT activity or the browning of WAT in humans has been linked to cold tolerance, enhanced energy expenditure, and protection against metabolic diseases, such as obesity and T2DM (for review Harms and Seale, 2013; Saito, 2013). In fact, the amount of metabolic active BAT in humans positively correlates with RMR and inversely with body mass index (BMI), fat mass (van Marken Lichtenbelt et al., 2009), and the development of T2DM. In addition, it has been shown that genetic variants of UCP_1_ are associated with fat metabolism, obesity, and diabetes. Among the UCP_1_ polymorphisms, the A3826G SNP in the promoter region has been associated with obesity, weight gain, and resistance to weight loss (reviewed in Jia et al., 2010).

## Uncoupling protein-3 (UCP_3_)

UCP_3_ is primarily expressed in skeletal muscle, but it is also found in BAT and heart tissue. Although UCP_3_ was first discovered and described in 1997 (Boss et al., 1997), the mechanisms of activation as well as its physiological role is still under debate (Cioffi et al., 2009). Due to its close homology with UCP_1_, UCP_3_ was initially implicated in thermoregulation (Boss et al., 1997), as it has been demonstrated to uncouple in a number of experimental models. However, other evidence has questioned the uncoupling activity of UCP_3_, including findings that (i) higher expression of UCP_3_ is not always associated with increased mitochondrial uncoupling (such as during fasting Cadenas et al., 1999) and (ii) UCP^−/−^_3_ mice do not show thermoregulation problems and are not obese (Gong et al., 2000; Vidal-Puig et al., 2000). Indeed, UCP_3_ mediated-uncoupling (called mild uncoupling) seems to be activated by specific cofactors, with FFA and reactive oxygen species (ROS) playing crucial and interrelated roles (Brand and Esteves, 2005; Lombardi et al., 2008, 2010), being the absence of one on these cofactors able to musk UCP3-mediated uncoupling.

UCP3 is also thought to play key regulatory roles in mitochondrial fatty acid oxidation and in preventing mitochondrial ROS-induced oxidative damage (Cioffi et al., 2009). Regarding the former, studies relating to the enhancement in SkM UCP3 expression have provided evidence for a close relationship to situations in which there is increased fatty acid oxidation (reviewed in Bezaire et al., 2007; Cioffi et al., 2009). SkM mitochondria isolated from UCP^−/−^_3_ mice have a lower ability to oxidize fatty acids than those from UCP^+/+^_3_ mice (Costford et al., 2008; Senese et al., 2011), that plausibly is responsible for the greater fat storage observed following long-term high fat feeding in mice lacking UCP_3_ (Nabben et al., 2011).

UCP_3_ also plays an important role in protecting cells against oxidative damage (Brand and Esteves, 2005; Goglia and Skulachev, 2003; Nabben et al., 2008; Schrauwen and Hesselink, 2004b). UCP_3_ may aid in mitigating ROS emission from the ETC; this role seems to be related to UCP_3_-mediated uncoupling and the consequent reduction in Δ*p*. In fact, Δ*p* is a key factor in influencing mitochondrial O^2−^ release (Korshunov et al., 1997) and a slight reduction in Δ*p* is associated to a significant depression of O^2−^ release. Interestingly, ROS itself or ROS by-products can induce UCP_3_ uncoupling, which provides a negative feedback loop for mitochondrial ROS production (Brand and Esteves, 2005). The sequential molecular mechanisms underlying ROS induced- and UCP_3_-mediated uncoupling seems to involve glutathionylation of the protein: a slight increase in ROS production promotes UCP_3_ de-glutathionylation, which activates UCP_3_-mediated uncoupling and further decreases ROS emission (Mailloux et al., 2011).

Another hypothesis concerning the role of UCP_3_ in protecting mitochondria from oxidative damage suggests that UCP_3_ is involved in the export of LOOH, which accumulates on the matrix side of the mitochondrial inner membrane (MIM), out of the matrix. This mechanism would reduce or eliminate LOOH from the inner leaflet of the MIM, which could otherwise trigger a cascade of oxidative damage to mitochondrial DNA and enzymes as well as other critical mitochondrial matrix-localized components (Goglia and Skulachev, 2003). This hypothesis has been validated by studies on mitochondria isolated from UCP^+/+^_3_ and UCP^−/−^_3_ mice, which underscores the ability of UCP_3_ to translocate LOOH and mediate LOOH-dependent mitochondrial uncoupling (Lombardi et al., 2010).

## Role of UCP_3_ in energy metabolism

Many studies have demonstrated that mitochondrial functionality is compromised in obesity and T2DM. In skeletal muscle, decreased mitochondrial uncoupling together with impaired mitochondrial fatty acid oxidation lead to fatty acids and/or their metabolites accumulation which, associated to increased oxidative stress, are crucial aspects in the development and progression of the above pathologies (Goodpaster et al., 2001; Patti and Corvera, 2010). Indeed, states of insulin-resistance are characterized by increased LOOH levels in SkM, and patients with T2DM exhibit damaged mitochondria with reduced functional capacity.

**Figure 2:**
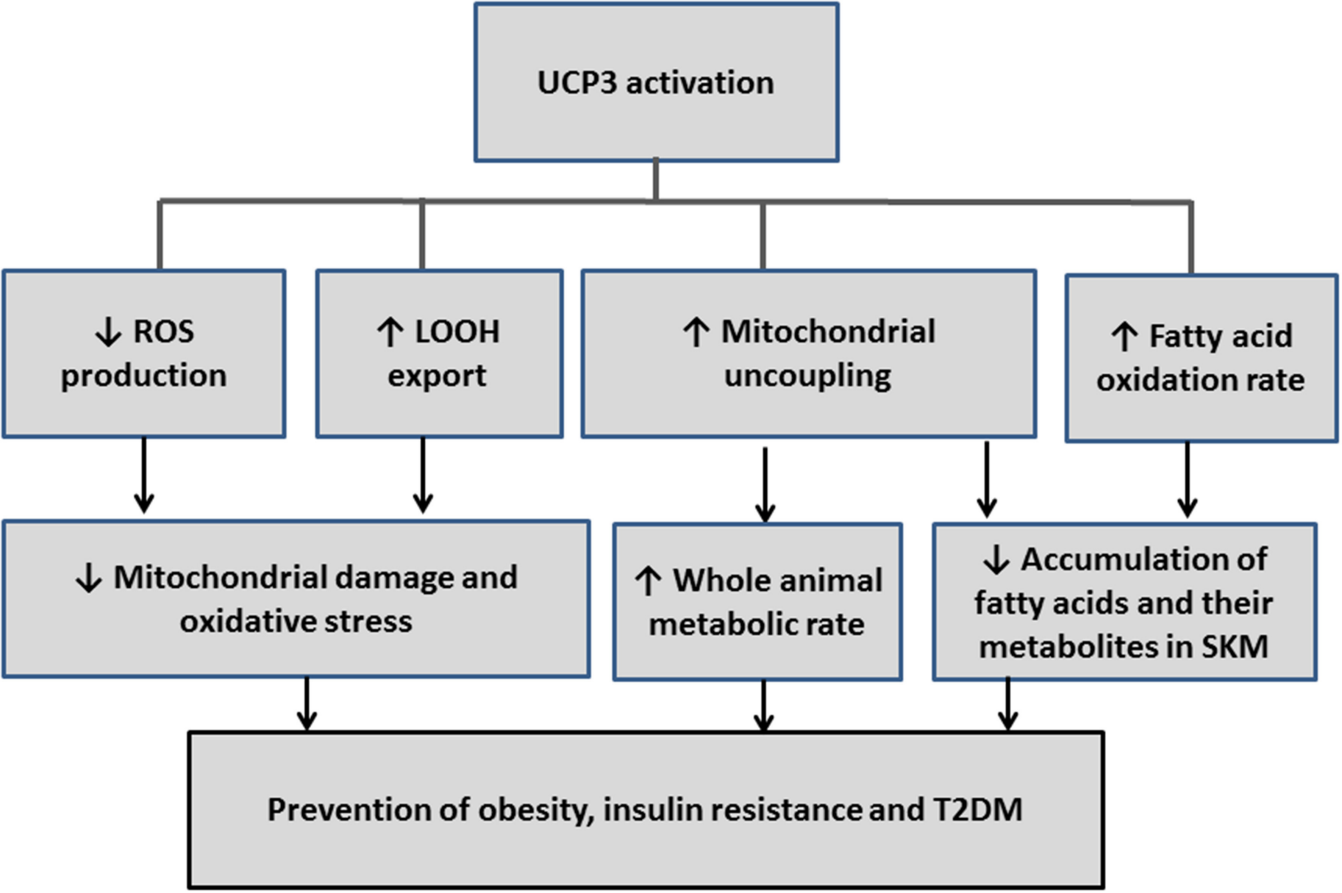
**Schematic diagram that illustrates how the reactions influenced by UCP3 could be redirected to prevent or treat obesity, insulin resistance, and type 2 diabetes (T2DM)**.

The involvement of UCP_3_ in mild uncoupling, in protection from ROS- and/or LOOH-induced oxidative stress, and in the amelioration of the fatty acid oxidation rate (see above) suggest a protective role for this protein in obesity and T2DM (Figure 2). This possibility is strengthened by findings that UCP_3_ is expressed in SkM, which is a tissue that represents 40% of metabolic active mass and largely contributes to energy homeostasis.

More experimental evidence supports a role of UCP_3_ in EM, including the findings that (i) UCP_3_ transgenic mice are less efficient in metabolism than wild-type controls (Costford et al., 2006) and are protected against high fat diet-induced obesity (Son et al., 2004; Costford et al., 2006); (ii) an enhancement of SkM UCP_3_ levels (also associated with higher BAT UCP_1_ levels) has been observed in obesity-resistant mice compared to obesity-prone mice (Fink et al., 2007); and (iii) modest UCP_3_ overexpression in skeletal muscle mimics physical exercise by increasing spontaneous activity and energy expenditure in mice (Aguer et al., 2013). Conversely, studies performed on UCP^−/−^_3_ mice argue against a role for UCP_3_ in EM, since these mice do not present reduced RMR and are not obese (Vidal-Puig et al., 2000). However, these studies were performed in mice housed at 20–22°C, a condition that, as described above, represents chronic thermal stress [see Uncoupling Protein-1 (UCP_1_) section]. Therefore, the occurrence of compensatory thermogenic mechanisms in UCP^−/−^_3_ mice could have masked the possible role of the protein in EM.

UCP_3_ also shows a protective role in T2DM. However, when studies were performed on transgenic mice, a clear involvement of the protein was evident when UCP_3_ overexpressing animals were used as a model (Choi et al., 2007; Costford et al., 2008). Instead, in UCP^−/−^_3_ fed a high fat diet regimen (known to induce a state of insulin resistance), insulin sensitivity has been reported to be unaffected (Vidal-Puig et al., 2000), enhanced (Costford et al., 2008), or reduced (Costford et al., 2008; Senese et al., 2011), with the effect being dependent on the age of the mice. Costford et al. (2008). On the other hand, UCP3^+/−^ heterozygous mice show an approximate 50% reduction in SkM UCP_3_ protein levels when compared to UCP3^+/+^, and a clear decline in insulin sensitivity has been reported. The effect was observed both when mice were fed with a standard diet or with a high fat diet (Senese et al., 2011). These data are in good accordance with clinical observations reporting that (i) a 50% reduction of UCP_3_ protein in human SkM is correlated with the incidence of T2DM (Schrauwen et al., 2001); and (ii) in humans, UCP_3_ protein levels are reduced in the pre-diabetic state of impaired glucose tolerance (Schrauwen and Hesselink, 2004a; Mensink et al., 2007).

Human Polymorphisms in the UCP_3_ gene also suggest an impact of UCP3 on fat metabolism, obesity, and T2DM. In this context, the UCP_3_ gene polymorphism –55T allele has been linked to enhanced UCP_3_ mRNA expression and RM (Schrauwen et al., 1999). Heterozygous C/T is associated with decreased obesity and T2DM risk (Meirhaeghe et al., 2000; Herrmann et al., 2003; Liu et al., 2005). Importantly, these findings have been recently confirmed through a meta-analysis showing an association of the polymorphism with protection from obesity in a European patient population (de Almeida Brondani et al., 2014). The association between the −55CT polymorphism of UCP3 and a lower BMI, however, is modulated by energy intake, since it disappears when caloric intake is increased (Lapice et al., 2014).

Heterozygous individuals that have a missense G304A polymorphism show decreased whole body fat oxidation compared to controls (Argyropoulos et al., 1998). They also present reduced levels of plasma lactate (Adams et al., 2009), that can be plausibly due to an impaired consumption of long chain fatty acids for muscle energy and to a greater reliance upon carbohydrates for energy.

Recently, four novel and heterozygous mutations in the UCP3 gene were identified (V56M, A111V, V192I, and Q252X) (Musa et al., 2012). Children carrying these mutations exhibited a higher percentage of fat and BMI, which was associated with dyslipidemia and lower insulin sensitivity (Musa et al., 2012).

## Concluding remarks and perspective

Targeting processes that lead to a reduction in mitochondrial coupling/efficiency and ROS production (thus oxidative stress) could be a promising therapeutic strategy to combat obesity and its co-morbidities (such as T2DM). The uncoupling proteins have several hypothesized functions including thermogenesis in certain tissues, protection from ROS, mediation of fatty acids oxidation and export of fatty acids, which are all related to the above pathologies. Hence, an understanding of the mitochondrial processes that lead to uncoupling and, in particular, the elucidation of the exact role played by UCPs at the mitochondrial level and in EM may provide an attractive therapeutic target for diseases rooted in metabolic imbalance.

In this context, the recruitment of thermogenic brown and/or beige adipocytes through activation of UCP_1_, and by expanding the tissue, through drugs or other methods, provide a promising approach for enhancing energy expenditure and combating obesity comorbidities. Recent evidence, indicating that brown and beige adipocyte can be recruited by different stimuli, raises the possibility that they may represent separate and distinct targets for therapeutic intervention. Although the roles of UCP_3_ are not completely clear, current data in human subjects (living at thermoneutrality) suggest a possible involvement of this protein in EM as well as in counteracting obesity, insulin resistance, and T2DM (Figure 2). Therefore, UCP_1_ and UCP_3_ represent promising therapeutic targets for treating pathologies that result from energy unbalance.

### Conflict of interest statement

The authors declare that the research was conducted in the absence of any commercial or financial relationships that could be construed as a potential conflict of interest.
